# Nutrient-rich spatial refuges buffer against extinction and promote evolutionary rescue in evolving microbial populations

**DOI:** 10.1098/rspb.2024.2197

**Published:** 2024-12-11

**Authors:** Matthew Kelbrick, Andrew Fenton, Stephen Parratt, James P. J. Hall, Siobhan O'Brien

**Affiliations:** ^1^ Department of Evolution, Ecology & Behaviour, University of Liverpool, Liverpool, UK; ^2^ Department of Microbiology, School of Genetics and Microbiology, Trinity College Dublin, Dublin, Ireland

**Keywords:** evolutionary rescue, ecological rescue, spatial refuges, motility, *Pseudomonas fluorescens*

## Abstract

Microbial populations are often exposed to long-term abiotic disturbances, which can reduce population viability and cause local extinction. Eco-evolutionary theory suggests that spatial refuges can facilitate persistence and evolutionary rescue. However, one drawback of spatial refuges is reduced exposure to nutrients such as carbon and oxygen, suggesting the protective effect of refuges depends on the interplay between environmental conditions and the degree of stress. Here, we test this general idea using mathematical modelling, and experimental evolution of the model bacterium *Pseudomonas fluorescens* SBW25 under salinity stress. As our model predicted, we find that the ability of spatial refuges to rescue evolving populations from extinction crucially depends on nutrient availability. Populations evolving under salinity stress where nutrient-rich spatial refuges were available, harboured clones that displayed enhanced salt resistance, indicating that nutrient-rich spatial refuges can facilitate evolutionary rescue. Furthermore, while control-salinity-evolved populations adapted to spatial structure by evolving enhanced motility (likely through parallel mutations in *PFLU_4551*, a predicted aerotaxis response regulator), this phenotype was constrained under high salinity, because increased motility negates the benefits of a spatial refuge. Our results reveal a general interplay between spatial refuges and nutrient availability that could be leveraged to reduce extinction risk in natural populations.

## 1. Introduction

The stability and resilience of microbial communities underpin fundamental ecosystem services—ranging from biogeochemical cycling to bioremediation [1,2]. However, microbial populations are increasingly subject to prolonged periods of directional change in the environment. As climate change and anthropogenic activities impose long-term disturbances on natural ecosystems, a major question is whether local populations can sustain themselves as conditions deteriorate [3–5]. If the environment deteriorates to the point at which population absolute fitness becomes negative, the population is on track for extinction [6]. However, since microbes evolve on rapid time scales, adaptation by natural selection could feasibly rescue populations from extinction—a process known as evolutionary rescue [6–8].

Environmental heterogeneity is a key facilitator of evolutionary rescue, by creating temporal or spatial refuges against an abiotic [9–12] or biotic [13,14] stressor [15]. For example, growing bacterial populations in static versus mixed cultures conditions can allow greater persistence of bacterial hosts against a biotic stressor (bacteriophage), because of reduced encounter rates under static conditions creating ephemeral refuges from bacteriophage infection [14]. However, many disturbances in natural environments are chemical—including pesticides, antibiotics and toxic metals such as arsenic and mercury, and increased salinity due to climate change. Under chemical stress, spatial refuges can similarly reduce encounter rates between bacteria and a chemical stressor, creating pockets of low-stressor refuges, such as via biofilm formation, auto-aggregation or cell-clustering [16–19]. Populations occupying refuges can recolonize the environment once a stressor is removed, increasing population stability against a disturbance [20]. Spatial refuges can act as key ‘hotspots’ for evolutionary rescue (i.e. *de novo* evolution and spread of stress-resistant phenotypes [11,12,21]) provided the strength of selection within the spatial refuge is sufficiently high.

While spatial refuges can reduce exposure to abiotic or biotic stressors, one drawback is reduced exposure to vital nutrients such as carbon, nitrogen and oxygen [10,19,22]. If nutrient acquisition becomes more limiting than the protection spatial heterogeneity provides, selection may act on genes that eliminate spatial structure (e.g. selection for increased motility or reduced biofilm formation). In other words, hiding from a stressor could potentially become costly when access to nutrients becomes more growth-limiting than the disturbance itself.

To investigate how nutrient availability and spatial heterogeneity affect evolutionary rescue, we first constructed a generalized mathematical model, which predicts how the benefits of spatial refuges are shaped by nutrient availability and increasing levels of abiotic stress. We then test these general predictions by way of experimental evolution of the plant growth-promoting rhizobacterium *Pseudomonas fluorescens*. We established a scenario where populations are exposed to either an abiotic stressor (high salinity) or not (control), under high and low nutrient conditions, and under well-mixed (homogenous) or static (heterogenous) conditions, with the latter creating conditions for spatial refuges (see [23]). We monitored population survival over time to assess extinction risk for each treatment combination. We then determined whether evolutionary rescue (salt adaptation) occurred in surviving populations, using a mixture of phenotypic assays and whole genome sequencing.

## 2. Methods

### (a) Theory

We developed a non-system-specific generalized mathematical model to explore the combined effects of nutrient limitation and an abiotic stressor (such as high salinity) on population persistence, in environments where spatial refuges could or could not form (manipulated by mixing). We modelled the abundance of a bacterium, *N*, which grows by feeding on nutrients at rate *R* and is suppressed by exposure to an abiotic stressor at rate *s*. Population growth is also limited due to intraspecific competition of strength *q*. To incorporate the effects of spatial structure, we assume a mixing rate, *µ*, which has a dual effect on the population: (i) mixing may circulate nutrients, which could boost bacterial growth, but (ii) mixing may also increase exposure to the stressor by removing spatial refuges, inhibiting population growth. The entire system is then described by the equation (2.1):


(2.1) $$\frac{dN}{dt}=\;N\left[\left(R+\mu \right)\left(1-Nq\right)-\left(s+\mu s\right)\right].$$

With this model, we explore the consequences of nutrient availability (*R*), abiotic stressor lethality (*s*) and spatial structure (mixing; *µ*) on population growth and persistence. We note that while our model explicitly describes nutrient availability as affecting growth rate rather than carrying capacity, and incorporates the effect of the stressor as a death term rather than as a modifier on growth rate, our conclusions are robust to such modifications (electronic supplementary material, text 1). Model analysis was carried out in Mathematica v. 12.1 [24].

### (b) Experiments

#### (i) Experimental evolution

We used *P. fluorescens* SBW25, modified to contain a streptomycin-resistant cassette and a lacZ insert for experimental evolution (SBW25-Sm^R^lacZ) [25,26]. The lacZ marker facilitated differentiation from an untagged ancestral clone during competition assays and had no effect on strain fitness when competed at 1 : 1 in LB media (*t*‐test: *t* = 0.324, *p* > 0.70). To begin the evolution experiment, a frozen stock culture of *P. fluorescens* was streaked onto lysogeny broth (LB) agar (Millipore) and incubated for 24 h at 28°C. Six single colonies were isolated using sterile inoculation loops and grown individually overnight in 6 ml LB (Luria; Millipore) at 28°C, shaking at 180 rpm. Each single-clone culture was used to inoculate one of six biological replicates per treatment in our fully factorial experimental design, where we manipulated salinity levels, nutrient availability and mixing.

To begin the evolution experiment, 10^4^ CFU ml^−1^ (equivalent for all treatments) of each *P. fluorescens* clone was used to inoculate 8 wells containing 200 µl LB in 96-well plates. For each clone, 4 of the 8 wells contained control-salinity LB (1% w/v NaCl), while the remaining 4 wells contained high-salinity LB (4.25% w/v NaCl). Control-salinity LB contained the baseline 1% salinity of LB, while high-salinity LB contained an additional 3.25% NaCl for a total of 4.25% (w/v). For each clone, half of the wells contained nutrient-rich growth media (standard LB), while the remaining 4 wells contained low-nutrient media. Nutrient limitation was established by diluting LB 1 : 10 with 1% or 4.25% saline to keep salt concentration consistent with the high/control salinity treatments [27]. Finally, we grew populations under either shaking (600 rpm) or static conditions; the latter imparted a relatively heterogeneous environment where spatial refuges could form [23]. For example, under static growth conditions, *P. fluorescens* SBW25 has been shown to acquire mutations and modify gene expression profiles, leading to increased biofilm formation at the air–liquid interface [22,28]. The refuges in this study are therefore not imposed by top-down design, rather we establish conditions under which spatial gradients of nutrients and stressors can form, and refuges can develop spontaneously (e.g. by cell aggregation, biofilm formation or migration) [29,30]. Overall, we employed a fully factorial experimental design where each set of conditions was replicated six times, and each replicate was initiated with an independent single clone of *P. fluorescens* (electronic supplementary material, figure S1). Populations were incubated at 28°C and 70% relative humidity for the duration of the experiment. After 24 h, populations were homogenized by pipetting and 2 µl (1%) of each population was transferred to 198 µl of the relevant fresh media in a 96-well plate using a sterile pin replicator. This was repeated every 24 h for 20 transfers, which is estimated to be 133 bacterial generations per transfer in the control treatment (20 × log2(100))—the number of generations may be less in stressor treatments. Frozen stocks were prepared every 5 days for all populations by mixing 150 µl of culture with 50 µl of 80% glycerol and storing at −80°C.

We assessed population densities on days 1, 5, 10, 15 and 20. Each population was serially diluted in LB before spreading 50 µl on LB agar using 5–10 sterile glass beads (5 mm; Witeg). Plates were incubated for 48 h at 28°C before colonies were counted. If no colonies were observed, 20 µl of each undiluted culture was plated out in triplicate. Populations were deemed to be extinct if no colonies appeared on plates from undiluted cultures.

#### (ii) Quantifying fitness of salt-evolved populations relative to their ancestor

**Figure 1.**
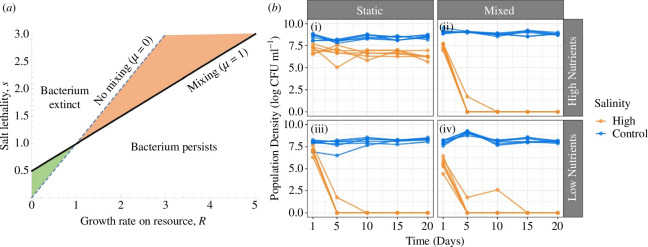
(*a*) Theoretical model of bacterial extinction and persistence under treatment conditions equation (2.1). Predicted effects of population growth, dependent on nutrient availability (*r*), stressor lethality (*s*) and population mixing (*μ*). The lines are boundaries between population persistence (below the lines) and population extinction (above the lines). The dashed line indicates the unmixed case (*μ* = 0); the solid line indicates the mixed case (*μ* = 1). The green region shows where population persistence is enabled by mixing due to increased resource availability when it would otherwise be driven extinct under static conditions. The orange region shows where the population is driven extinct by mixing due to increased stressor exposure. (*b*) Population densities (log_10_ CFU ml^−1^) were quantified throughout 20 days of an evolution experiment, where *P. fluorescens* was evolved under 8 different treatment conditions. We find that populations can only persist under high salinity (orange lines) when spatial refuges are allowed to form (i.e. static conditions), and nutrient availability is relatively high (panel (i)). See main text for statistics. Individual lines represent a single replicate population, with six replicates per treatment combination.

To investigate whether salinity-evolved populations increased fitness relative to the ancestor, and how this was in turn affected by whether competitions were performed at high versus control salinity, we performed head-to-head competition experiments [31,32]. We focused on the six populations where ecological rescue occurred (i.e. the population was buffered against extinction; high nutrients, static, day 20; figure 1*b*(i), orange). We competed our SBW25 reference ancestral strain (no LacZ marker) against each evolved population (harbouring a lacZ marker) in a 1 : 1 ratio under high- and control-salinity levels. Competitions were performed in 96-well plates containing 180 µl LB, which was amended to a final salinity of either 4.25% (w/v) NaCl (high salinity) or 1% (w/v) NaCl (control-salinity). Six evolved populations were competed against ancestral SBW25, and each competition was replicated six times. Starting densities of each competitor were standardized to OD 0.1 (600 nm) (~10^8^ CFU ml^−1^) and serial diluted to 10^−4^ before adding 10 µl of each competitor to relevant wells. Plates were incubated statically at 28°C for 24 h. Starting and final densities (CFU ml^−1^) were enumerated for each competitor by spreading samples of mixed cultures on LB agar containing 50 μg ml^−1^ X-Gal and incubating for 48 h at 28°C. Evolved colonies were distinguished from ancestral colonies via the formation of blue-pigmented colonies due to the breakdown of X-gal by lacZ-encoded beta-galactosidase.

#### (iii) Quantifying salt resistance of single colonies isolated from surviving high-salinity-evolved populations

To investigate whether high-nutrient spatial refuges facilitated evolutionary rescue through increased resistance in high-salinity environments, we selected at random nine colonies from each of the six high-salinity-evolved populations that survived the duration of the experiment (i.e. high nutrients, static, day 20; figure 1*b*(i), orange). As a control, we also selected nine colonies from each of the six control-salinity-evolved populations evolving under the same ecological conditions (i.e. high nutrients, static, day 20; figure 1*b*(i), blue). Whole populations were diluted and plated on LB agar, incubated for 72 h at 28°C, before 9 colonies were selected from each of the 12 populations. Colonies were grown for 24 h individually in a 96-well plates containing 200 µl LB. After 24 h, populations were diluted × 100-fold with either high-salinity LB (4.25% NaCl) or control-salinity LB (1% NaCl), before 20 µl of diluted culture was added to either 180 µl control-salinity LB or high-salinity LB. Hence, the growth of all 108 clones was determined under both high and control salinity. Each assay was replicated three times per clone. The growth of six ancestral clones used to initiate the evolution experiment was also quantified under high and control salinity; these assays were replicated six times. Plates were incubated statically at 28°C for 24 h. Optical density (OD) at 600 nm was measured at the start and end of the experiment using a Tecan Nano plate reader.

#### (iv) Resequencing methods and bioinformatic analysis

**Figure 2.**
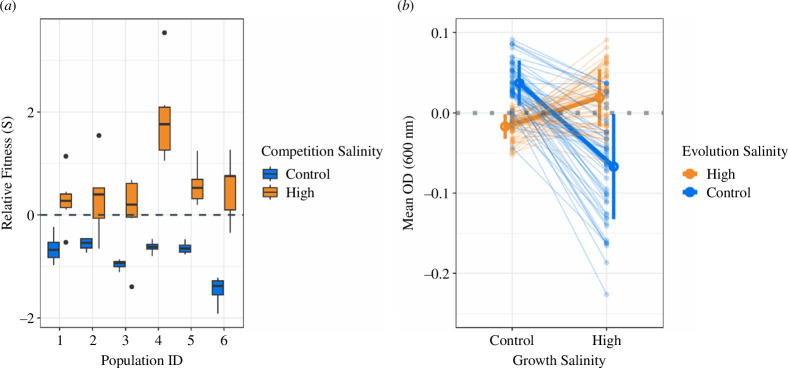
(*a*) Relative fitness (S) of high-salinity evolved populations against ancestor, under control or high-salinity conditions. When *S* = 0, the fitness of both strains is equal. When *S* > 0, evolved strains outcompete the ancestor. Error bars show 95% confidence intervals. *n* = 6. (*b*) Optical densities (OD_600_) after 24 h growth in control or high salinity environments for 9 clones per 12 evolved populations (6 high-salinity and 6 control-salinity evolved). Light-coloured individual lines represent the growth of individual clones and thicker lines show means and standard deviations across all 54 clones per evolution treatment and growth salinity condition combination. OD_600_ values are calculated relative to the population ancestor; i.e. when *y* = 0, no changes in salt resistance are observed between evolved and ancestral clones. Clones evolved under high-salinity have increased growth relative to ancestor when grown under high-salinity conditions, and worse growth relative to ancestor when grown under control-salinity conditions. Conversely, clones evolved under control-salinity have improved growth relative to ancestor when grown under control-salinity conditions, but worse growth relative to ancestor when grown under high-salinity conditions. See main text for statistics.

To identify potential genetic drivers of evolutionary rescue (i.e. salt adaptation) in evolved clones, we performed whole genome resequencing on the three most salinity-resistant clones per six high-salinity evolved populations identified in §2b(iii). To test whether any observed parallel mutations in these clones were treatment-specific, we also sequenced the three most salinity-resistant clones from each control-salinity-evolved population, which evolved under the same ecological conditions (figure 2*b*). The six ancestral clones used to initiate each replicate population were also sequenced.

Clones were grown for 48 h on LB agar. Once grown, biomass was scraped from plates using a sterile inoculation loop and suspended in tubes containing 500 µl of DNA/RNA shield inactivation buffer (MicrobesNG). MicrobesNG performed genomic DNA extraction and whole genome sequencing using Illumina NovaSeq 6000 (2 × 250 bp; 30 × coverage). The bioinformatical services of MicrobesNG trimmed adapters using Trimmomatic version 0.30 [33]. Variant calling was performed using the breseq computational pipeline (version 0.37.1 [34]) and the SBW25 reference sequence (accession: AM181176), giving a dataset containing 78 mutations. To accommodate mutations in our laboratory strain relative to the reference genome, and to control for spurious prediction of mutations in repetitive or difficult-to-map parts of the reference, any mutations identified in our 6 ancestral clones relative to the SBW25 reference sequence, including mutations with ‘marginal’ or ‘unassigned’ evidence, were pooled and removed from all clones in the evolved variant dataset prior to statistical analysis; this removed 43 unique mutations. The remaining 35 mutations comprised gene deletions (31.4%), insertions (11.4%) and single nucleotide polymorphisms (57.1%; SNP)—including non-synonymous (42.8%), synonymous (2.8%), intragenic (8.6%) and nonsense (2.8%) (electronic supplementary material, table S1). Evolved clones had between 0 and 5 mutations with a median of one mutation per clone (electronic supplementary material, table S1). All reads have been uploaded to the NCBI SRA database (BioProject ID: PRJNA1142763).

#### (v) Motility assays

To assess swimming and swarming motility, we selected one sequenced clone containing a mutation in PFLU_4551 from each control-salinity evolved population (high-nutrients, static, day 20; figure 1*b*(i), blue). We also selected single clones from each high-salinity population that evolved under the same conditions (high-nutrients, static; day 20; figure 1*b*(i), orange)—all these clones lacked the PFLU_4551 mutation. These 12 clones (alongside a positive control of a motility deficient *ΔgacS* mutant [35,36] were grown in 50 ml falcon tubes containing 5 ml of LB and incubated shaken at 180 rpm for 24 h at 28°C. Semi-solid agar was made using LB containing 0.3% or 0.6% (w/v) agar for swarming or swimming assays, respectively. Agar plates were poured and left for 1 h to dry, then 10 µl of overnight culture standardized to an OD 600 nm of 0.2 was pipetted just below the agar surface at the centre of the plate and incubated upright at 28°C. Swimming halo diameters were measured after 24 h using a ruler. Swarming ability was measured from the inoculation site to the point of furthest growth. All assays were performed in triplicate.

#### (vi) Statistical analysis

The effect of evolutionary treatment on *P. fluorescens* densities after 24 h was calculated using a three-way ANOVA. For control-salinity evolved populations, we assessed how population densities changed over time through maximum likelihood analysis using a linear mixed-effects model (LMM) using the package ‘nlme’ [37], assigning ‘nutrient-level’ (factor), ‘spatial structure’ (factor) and ‘time’ (numeric) as explanatory variables (including a three-way interaction) and ‘time’ and ‘replicate’ as random effects to account for repeated measures. The significance of fixed effects was determined by likelihood ratio tests.

To quantify the relative fitness of high-salinity evolved populations relative to the ancestor, the relative fitness (S) of evolved populations was calculated as the ratio of Malthusian parameters: *S* = ln(^evolved^_end_/^evolved^_start_) − ln(^ancestor^_end_/^/ancestor^_start_) [38]. We tested how *S* was affected by competition under high versus control-salinity using a one-way ANOVA. We tested whether the growth of individual clones isolated from high- and control-salinity evolved populations was affected by evolutionary history (i.e. high- versus control-salinity) and salt exposure using LMM, with ‘evolutionary history’ (factor) and ‘salinity treatment’ (factor) as explanatory variables (including their interaction), plus ‘population/clone’ as nested random effects. The significance of the interaction was determined by a likelihood ratio test. A one-sample *t*‐test was used to compare the mean growth of evolved clones (CFU ml^−1^) to ancestral growth (*y* = 0) under the same salinity-growth condition.

The number of non-synonymous and synonymous mutations was calculated for the control and high-salinity treatments using gdtools ‘UNION’ and ‘COUNT’ on the breseq output files [34]. To detect if mutational patterns were associated with treatment, we performed a permutational MANOVA using the ‘adonis’ function from the ‘vegan’ v 2.6-4 package [39].

For the motility assay, evolved clone motility was standardized against the relevant ancestor (i.e. ancestral motility = 0), and the differences in swimming and swarming motility based on the evolutionary treatment were determined by one-way ANOVA. A Bonferroni-corrected one-sample *t*‐test was used to collectively compare all high- and control-salinity clone motility against the ancestor. Analyses were performed using R v. 4.2.3 [40].

## 3. Results

### (a) Theoretical predictions

To predict how spatial refuges and nutrient availability may jointly impact population persistence in the presence of an abiotic stressor (e.g. high salinity), we developed a generalised extension to the standard logistic growth model, to incorporate an additional ‘mixing’ factor, *µ*, describing the joint effect of spatial heterogeneity on both nutrient availability and exposure to an abiotic stressor such as salt equation (2.1). Our model predicts two stable states: (i) population extinction ($N^{*}=0$), or (ii) population persistence at an equilibrium level $N^{*}=R+\mu -s\left(1+\mu \right)/q\left(R+\mu \right)$, which is increased by additional resources (R) and reduced by the abiotic stressor (s). The eigenvalue of the system at the first state is R+μ-s(1+μ) and the eigenvalue at the second state is -R-μ+s(1+μ), showing these two states have mutually exclusive stability, and are separated by a boundary given by R=μ(s-1)+s. In the absence of mixing (μ=0), high stressor lethality (high *s*) will drive population extinction, while high nutrients (high *R*) facilitate persistence. Mixing, however (μ>0), shifts the balance of these opposing effects (figure 1*a*, solid line, shown for μ = 1). When nutrients are limited (low N), mixing can allow the population to persist by making additional resources available, but only if stressor lethality is low (figure 1*a*, green region). When stressor lethality is high, the positive effect of mixing on resource acquisition is negated by increased exposure to the stressor (figure 1*a*, orange region).

### (b) Experiments

#### (i) Experimental evolution: the ability of spatial refuges to prevent population extinction depends on nutrient availability

To test the predictions of our model experimentally, we used experimental evolution of *P. fluorescens*, where replicate populations were cultured under high versus control salinity, high versus low nutrient availability, and in the presence versus absence of spatial structure. We manipulated the presence versus absence of spatial refuges, by growing populations either static or mixed, respectively [23]. We first quantified the reduction in population density after 24 h caused by each experimental condition independently and in combination. As predicted, while nutrient reduction and salinity reduced population size after 24 hours growth, changes in spatial structure (i.e. agitation) had a significant three-way interaction with the other treatments, such that disruptions to spatial structure significantly reduced population sizes relative to expectations when populations were faced with reduced nutrients and increased salinity together (ANOVA: three-way interaction effect of agitation × salinity × nutrient: F_1,40_= 6.757, *p* < 0.02).

Next, we tested how high salinity, nutrient limitation and static growth affected population dynamics during our 20 day evolution experiment. As expected, in control-salinity treatments, all populations remained viable throughout the entire 20 days of the experiment (figure 1*b*(i), blue), reaching higher densities when evolving under high versus low nutrient conditions (LMM, nutrient level: χ^2^ = 47.680, *p* < 0.01), and grown under mixed versus static conditions (LMM, spatial structure: χ^2^ = 31.799, *p* < 0.01), irrespective of timepoint. On the other hand, 18/24 populations evolving under high salinity were driven extinct by the final timepoint (figure 1*b*(ii),(iv), orange). The remaining 6/24 populations persisted at high densities (~10^6^) for the duration of the experiment; these populations were all grown under high nutrients and static conditions (figure 1*b*(i)). This finding aligns with our theoretical predictions, where persistence increased under high nutrient availability and without mixing, allowing nutrient-rich spatial refuges to buffer against extinction (figure 1*a*, orange region).

#### (ii) High-salinity populations equal or outcompete the ancestor under high salinity only

While spatial refuges prevented extinction in both our evolution experiment and our theoretical model under high nutrient conditions, it is unclear whether refuges can facilitate *de novo* adaptive evolution to a stressor or if minimizing exposure instead weakens selection for resistance. To investigate whether high-salinity evolved populations have increased resistance to salt compared to ancestral *P. fluorescens,* we competed surviving high-salinity evolved populations (i.e. figure 1*b*(i), orange, day 20) against ancestral *P. fluroescens* under both high and control-salinity. The relative fitness (S) of evolved populations versus the ancestor was greater when competitions were performed in high- versus control-salinity environments (ANOVA: F_1,70_ = 81.78, *p* < 0.01, figure 2*a*). However, this is largely driven by the loss of evolved population competitive fitness when competitions are performed in the absence of salt. Hence, high-salinity-evolved populations are less adapted to their environment relative to the ancestor under control salinity levels, but increasing salinity restores fitness to equal or greater than the ancestral growth.

#### (iii) Salt resistance data for individual clones indicate trade-offs in adapting to salinity versus experimental growth conditions alone

We quantified salt resistance for 108 individual clones that evolved under either control or high salinity. The growth (carrying capacity) of clones under high or control salinity was directly determined by their evolutionary history, i.e. the salinity treatment they evolved under (LMM: Salinity grown at × Salinity evolved at: χ^2^ = 124.594 *p* < 0.01, figure 2*b*). Specifically, high-salinity evolved clones, on average, grew slightly better than their ancestor under high salinity (*t*‐test: t = 3.8933, *p* < 0.02) but slightly worse than their ancestor at control salinity levels (*t*‐test: t = −8.0841, *p* < 0.01). In other words, while resistance to high-salinity is observed for high-salt evolved clones, these clones are less adapted to other aspects of their environment compared to the ancestor. The opposite is true for low-salinity evolved clones which grew worse than their ancestor under high salinity (*t*‐test: t = −7.5188, *p* < 0.01) but better under control salinity (*t*‐test: *t* = 9.7724, *p* < 0.01). This suggests a trade-off between evolved salt resistance and environmental adaptation, which may also drive the overall loss of population fitness observed in §3b(ii) (figure 2*a*). Furthermore, there is a large diversity in the apparent trade-off in salt resistance between individual clones (i.e. some clones have a large difference in OD, while other clones have a small difference in OD, when grown under high- and control-salinity), indicating that populations may have high genetic diversity—possibly due to their structured environment providing different niches or stress gradients.

#### (iv) Genomic responses to selection in evolving populations

**Figure 3.**
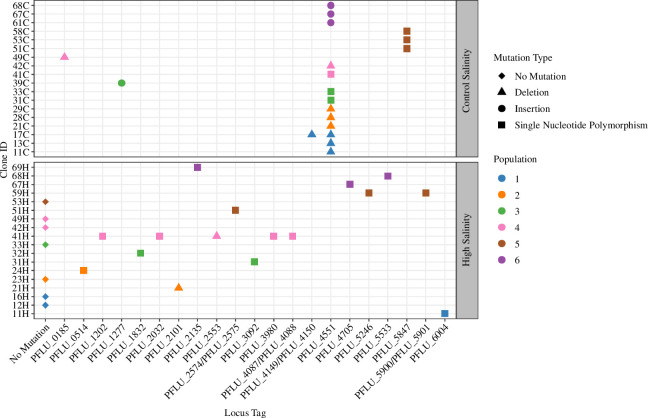
To investigate any genetic mutations driving evolutionary rescue, we selected 3 clones from each of 6 high and 6 control salinity populations at the final timepoint (*n* = 36) for whole genome resequencing (figure 1*b*(i), day 20). High-salinity-evolved clones contained a greater number of unique mutated loci compared to the control-evolved clones (unique loci targeted: control salinity = 5, high salinity = 16). We identified one loci (PFLU_4551) which was targeted by mutation in > 1 population, i.e. representing locus-level parallel mutations, which indicate natural selection acting upon a specific gene or genes [41]. Mutations in *PFLU_4551* were present in the majority of control-salinity evolved clones (13/18 clones from 5/6 populations). Points are coloured based on their population origin. Points shape indicates mutational type: diamond = no mutations; triangle = deletion; circle = insertion; square = single nucleotide polymorphism (SNP). Clone ID nomenclature is as follows: first number = population ID; second number = clone number; letter = salinity level (e.g. 67H = population 6, clone 7, high-salinity evolved).

To pinpoint genetic mutations driving salt adaptation, we selected 3 clones from each of 6 high and 6 control-salinity populations (*n* = 36) for whole genome resequencing. The spectra of loci disrupted by non-synonymous mutations differed between high and control salinity treatments (permutational MANOVA: effect of salinity *F* = 2.1429, *p* < 0.02; figure 3; electronic supplementary material, table S1), indicating that mutational patterns were treatment specific (unique loci targeted: control salinity = 5, high salinity = 16). Of these loci, one was targeted in > 1 population in one treatment and in 0 populations in the alternative treatment, i.e. representing a treatment-specific locus-level parallel mutation, which indicates natural selection acting upon a specific gene or genes [41] (figure 3). This locus was *PFLU_4551*, an orthologue of *aer*, a gene known to contribute to cellular motility and chemotaxis in *P. aeruginosa* [42]. Mutations in *PFLU_4551* were present in the majority of control-salinity evolved clones (12/18 clones from 5/6 populations) and were never present in clones evolved under high salinity (figure 3). Furthermore, the three control-salinity evolved clones without a mutation in *PFLU_4551* (51C, 53C and 58C) had similar growth to the ancestor under control-salinity conditions, while all PFLU_4551 clones had increased growth (electronic supplementary material, figure S3), indicating that PFLU_4551 mutants may enhance the growth of clones growing under control-salinity conditions. Mutations in *PFLU_4551* included in-frame insertions and deletions in a CCG trinucleotide repeat, a C>R non-synonymous mutation and a premature stop codon (at codon 193/522). We therefore hypothesized that loss-of-function mutations in *PFLU_4551* may be responsible for the observed increased population fitness in our experimental setup, but are constrained under high salinity.

#### (v) Mutations in PFLU_4551 are associated with increased swarming motility

**Figure 4.**
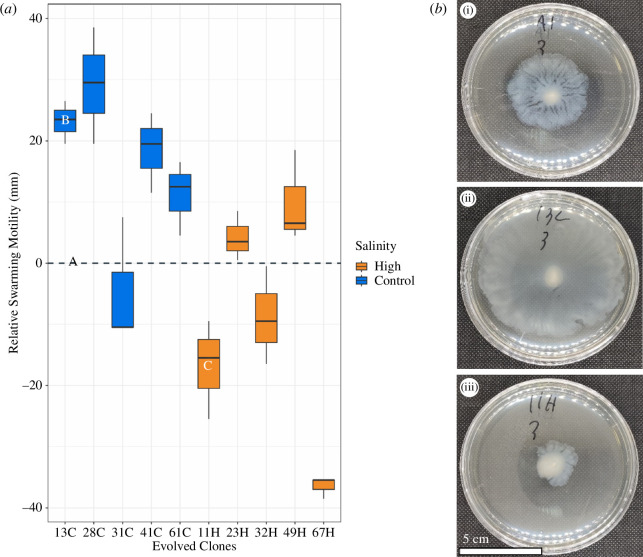
(*a*) Swarming motility of control-salinity evolved PFLU_4551 mutant clones (blue) and high-salinity evolved PFLU_4551 wildtype clones (orange). All motility results are calculated relative to their ancestral mean (when *y* = 0, motility is equal for ancestral and evolved clones). Box plots show means and 95% confidence intervals for 6 replicates per clone. Swarming motility was significantly higher for PFLU_4551 mutants compared to wildtype variants (see main text for statistics). Photos (right) corospond with points on the figure (left) containing the same lable (a,b, and c). Photos show swarming motility of: (a) ancestral, (b) clone 13C (PFLU_4551 mutation), and (c) clone 11H (wildtype PFLU_4551). Evolved clone nomenclature is as follows: first number = population ID; second number = clone ID; letter = salinity evolved under (e.g. 67H = population 6, clone number 7, high-salinity evolved).

Our mutational analysis identified PFLU_4551 as a target of selection in control salinity, but not high-salinity environments. *PFLU_4551* is an orthologue of *aer*, which has been shown to control chemotaxis and cell motility [43,44]. To investigate whether mutations in *PFLU_4551* are associated with altered motility, we performed a soft agar swarming and swimming motility assay. Swimming motility did not differ between PFLU_4551 mutants (all originating from control-salinity evolved populations) and wildtype variants (all originating from high-salinity evolved populations; electronic supplementary material, figure S3; ANOVA: *F* = 0.231, *p* > 0.5). However, swarming motility was significantly higher for PFLU_4551 mutants compared to wildtype variants (figure 4; ANOVA: *F* = 17.45, *p* < 0.01), and ancestral clones (*t*‐test: *t* = 3.9845, *p* < 0.01). In comparison, evolved clones with wildtype *PFLU_4551* displayed no significant change in swarming motility overall (*t*‐test: *t* = −2.2474, *p* > 0.05). Together, our findings support the idea that increased motility (via mutations in *PFLU_4551*) is advantageous in our spatially structured environment, but such adaptation is constrained under high salinity.

## 4. Discussion

Spatial refuges are an important ecological mechanism for preventing population extinction under abiotic stress and can facilitate evolutionary rescue [15,20]. However, spatial structure (an essential prerequisite for refuges) is itself a stressor, particularly when key nutrients are limiting [19,45]. Using theory and experiments, we show that spatial refuges can prevent extinction only when nutrients are available, and that only nutrient-rich refuges provide opportunities for evolutionary rescue. Moreover, we reveal a general trade-off between adapting to spatial structure alone (selection for increased motility) and adaptation to an abiotic stressor (selection for reduced motility and hence maintenance of a spatial refuge). Our competition experiments and salt resistance assays further support this trade-off: adaptation to high salinity conferred worse growth under control salinity conditions and vice versa. Together, our results indicate a general trade-off between spatial refuges and nutrient acquisition which in turn shapes the evolutionary dynamics of surviving populations.

We exposed *P. fluorescens* to multiple co-occurring stressors (nutrient limitation, spatial structure, and salt stress), which affected population eco-evolutionary dynamics differently depending on whether stressors were imposed individually or in combination. Studies on multi-stressor interactions, such as with phages and antibiotics, have shown that in some cases, exposure to one stress can increase resistance to the alternative stressor [46]. However, such a pattern is dependent on the nature of the stressors involved and exact mechanisms of resistance. For example, *P. fluorescens* populations that evolved in the presence of sublethal antibiotic concentrations and a predator *Tetrahymena thermophila* showed delayed adaptations to one or both stressors compared to the stressors individually [47]. Similarly, in our study, high salinity constrained adaptation to spatial structure because of motility-associated trade-offs. Furthermore, multiple stressors may constrain adaptation when population densities are greatly reduced—since lower densities reduce the likelihood of evolutionary rescue and increase extinction risk [6,8,48]. This was observed in our study, where nutrient limitation caused extinction of all populations under salinity stress, irrespective of whether spatial refuges could form.

Our resequencing analysis was unable to link the observed high-salinity resistance phenotype to a specific mutation. A lack of clear genetic signatures for salt adaptation may be because, unlike stressors such as antibiotics that have explicit modes of inhibiting bacterial growth (i.e. specific target binding sites [49]), stressors such as salt can impact microbes in numerous ways. For example, high salinity can: (i) destabilize the water potential across cell membranes causing cell lysis; (ii) induce the energy- and resource-expensive upregulation of membrane-bound ion and compatible solute transporters; (iii) increase the biosynthesis of compatible solutes; (iv) denature proteins (‘salting-out’) requiring *de novo* mutations encoding a higher proportion of hydrophilic amino-acid residuals to prevent salting-out [50]. While we were unable to pinpoint any locus-level parallel mutations in high-salinity evolved clones, we found that high-salinity evolved clones have a higher number of unique locus-level mutations (*n* = 16) compared to clones evolved under control conditions (*n* = 5). Thus, we suggest that adaptation to salinity in our salinity-evolved clones is likely driven by the collective outcome of mutations in a range of genes in different clones coding for different resistance mechanisms, similar to findings in *P. aeruginosa* [51]. Seven clones belonging to the high-salinity treatment showed no evidence of identifiable mutations, despite some clones growing better than their ancestor under high-salinity, suggesting that epigenetics or structural changes and mutations in repeat regions, which are difficult to detect by short-read sequencing, may be responsible for changes in salinity resistance [52].

We identified *PFLU_4551* as a target of selection in control-salinity evolved clones, present in 12/18 clones from 5/6 populations. *PFLU_4551* is a putative aerotaxis receptor and an ortholog for the *aer* gene in *P. aeruginosa*. The encoded protein, Aer, is a transmembrane aerotaxis receptor that allows organisms to sense oxygen gradients in their environment and alter their motility accordingly [42]. Mutations in *aer* alter cell swarming and swimming motility, and biofilm formation in *Pseudomonas putida* and *Pseudomonas protegens* [43,53,54]. In our study, *PFLU_4551* mutations caused enhanced swarming relative to ancestral and wildtype clones, further supporting this previously uncharacterized role of *PFLU_4551* in motility. Together, this supports a role for PFLU_4551 in swarming motility, which can increase nutrient access in a spatially structured environment [55,56]. Increased motility is likely under selection in our spatially structured populations, where O_2_ and nutrient gradients can influence the evolutionary trajectory of *P. fluorescens* [22].

Selection on motility-associated traits has important consequences for plant-growth-promoting bacteria (PGPB) such as *P. fluorescens*. Increased motility improves plant root colonization and biocontrol ability—suggesting adaptation to spatial structure even in our artificial lab environment could improve ecosystem functioning. Conversely, reduced motility in high-salinity evolved clones could reduce plant colonization ability and pest-suppression capabilities of this PGPB [57]. While we focus solely on one abiotic stressor here (salinity), it is possible that a combination of stressors imposed in harsh environments (such as intensive agriculture) could further select against motility in PGPB, reducing the protective effect PGPB has on plants as well as ecosystem services and crop productivity [58]. Furthermore, in soil ecosystems, intensive land management practices alongside climate change are increasing the influx of abiotic stressors (salinity, pesticides, antibiotics) [5], driving soil degradation [59–61] and causing a decline in soil nutrient content [62,63]. Our results suggest that these combined stressors could be a triple threat for soil microbial communities, diminishing the potential for evolutionary rescue and instead driving local extinction of susceptible bacteria.

## Data Availability

Genomes can be accessed at the BioProject ID: PRJNA1142763 Code is available at Zenodo [64]. Datasets are available at Dryad [65]. Supplementary material is available online [66].
